# Supplementary material for: Structural basis for human NKCC1 inhibition by loop diuretic drugs
Source: EMBO J. 2025 Jan 28;44(5):1540–62. doi: 10.1038/s44318-025-00368-6 (PMC11876703; doi:10.1038/s44318-025-00368-6)
Supplement: Supplementary file 1 — Appendix [file 44318_2025_368_MOESM1_ESM.pdf]

Appendix for

Structural basis for human NKCC1 inhibition by loop diuretic drugs

Yongxiang Zhao *et al.*

*Corresponding author: erhu.cao@biochem.utah.edu

This PDF file includes:

p2.....Appendix Figures S1
p4.....Appendix Figures S2
p5.....Appendix Figures S3
p7.....Appendix Figures S4
p8.....Appendix Figures S5
p10...Appendix Figures S6
p11...Appendix Figures S7
p13...Appendix Figures S8
p15...Appendix Figures S9
p17...Appendix Figures S10
p18...Appendix Figures S11
p19...Appendix Figures S12
p20...Appendix Figures S13
p21...Appendix Table S1

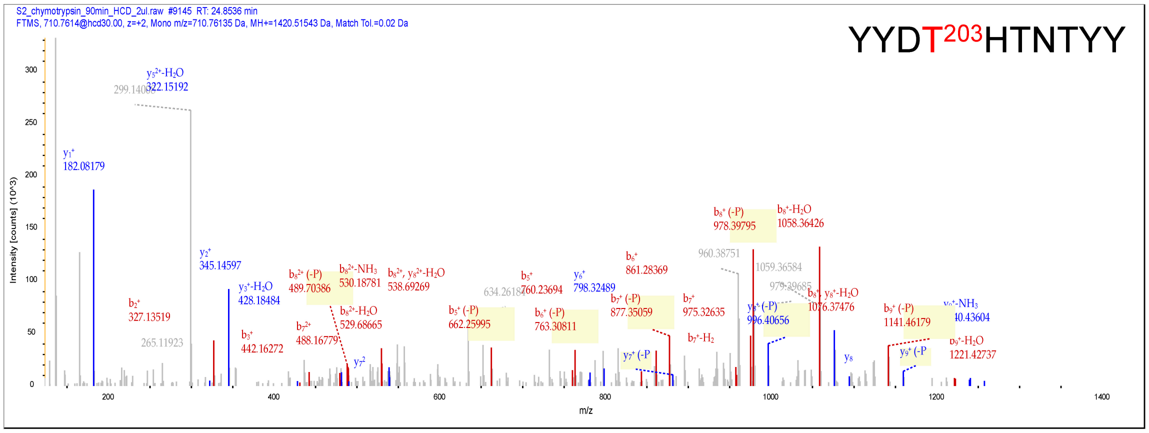
S2_chymotrypsin_90min_HCD_2ul.raw #9145 RT: 24.8536 min
FTMS, 710.7614@hcd30.00, z=+2, Mono m/z=710.76135 Da, MH+=1420.51543 Da, Match Tol.=0.02 Da
YYDT^{203}HTNTYY
Intensity [counts] (10^3)
m/z

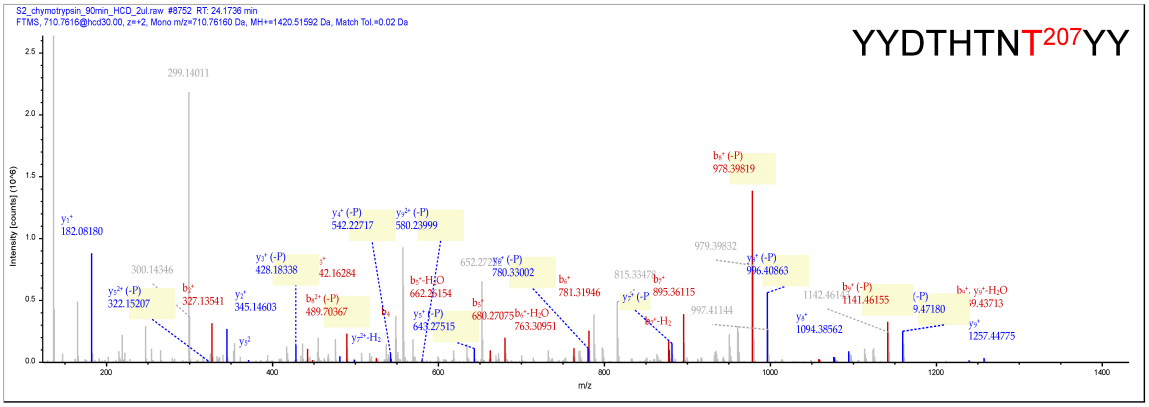
S2_chymotrypsin_90min_HCD_2ul.raw #8752 RT: 24.1736 min
FTMS, 710.7616@hcd30.00, z=+2, Mono m/z=710.76160 Da, MH+=1420.51592 Da, Match Tol.=0.02 Da
YYDTHTNT^{207}YY
Intensity [counts] (10^6)
m/z

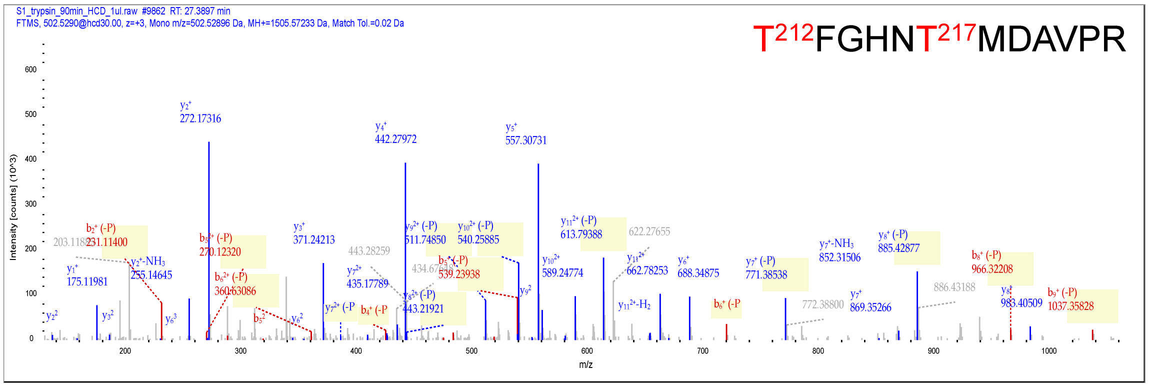
S1_trypsin_90min_HCD_1ul.raw #9862 RT: 27.3897 min
FTMS, 502.5290@hcd30.00, z=+3, Mono m/z=502.52896 Da, MH+=1505.57233 Da, Match Tol.=0.02 Da
T^{212}FGHNT^{217}MDAVPR
Intensity [counts] (10^3)
m/z

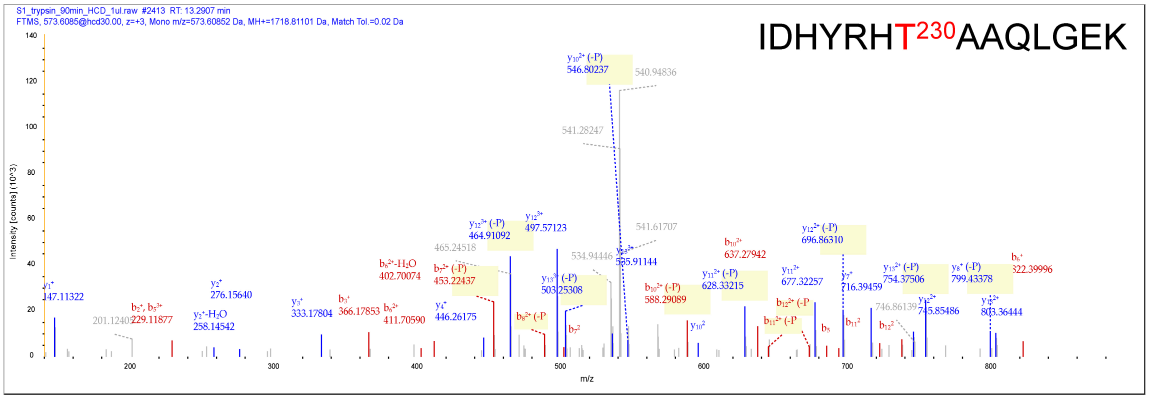
S1_trypsin_90min_HCD_1ul.raw #2413 RT: 13.2907 min
FTMS, 573.6085@hcd30.00, z=+3, Mono m/z=573.60852 Da, MH+=1718.81101 Da, Match Tol.=0.02 Da
IDHYRHT^{230}AAQLGEK
Intensity [counts] (10^3)
m/z

Appendix Figure S1. **Mass spectra of phosphorylation on Thr203, Thr207, Thr212, Thr217, and Thr230 of human NKCC1.** Human NKCC1 sample was purified from HEK293 cells that also co-express WNK1 and SPAK kinases. After digestion with either trypsin or chymotrypsin, samples were analyzed by a nanoUPLC-MS/MS system followed by proteomic data analysis. Phosphorylation on Thr203, Thr207, Thr212, Thr217, and Thr230 of NKCC1 was identified with high confidence (~ 100%).

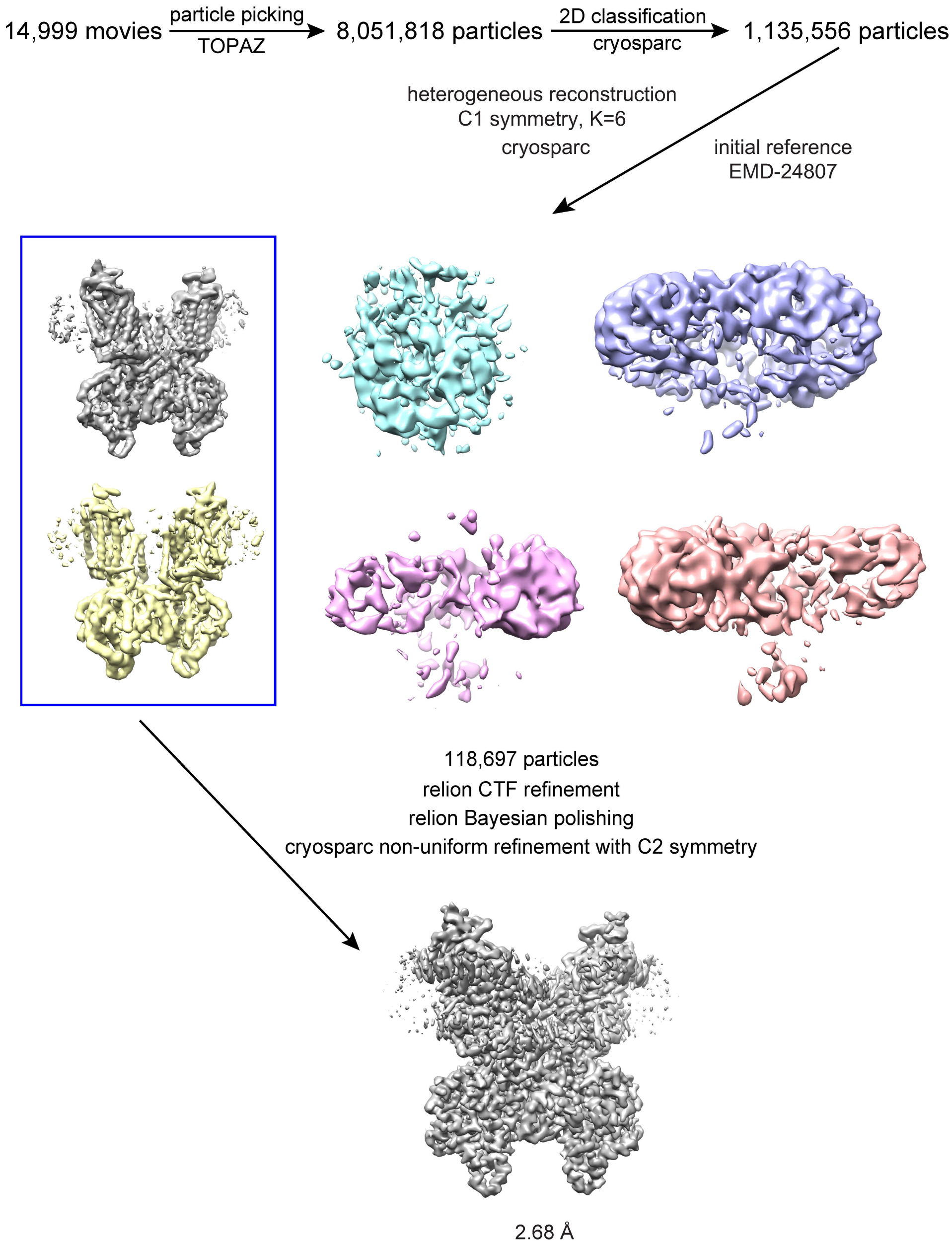

Appendix Figure S2. Reconstruction of human pNKCC1 bound with furosemide. Flow chart of image processing for the pNKCC1/furosemide dataset.

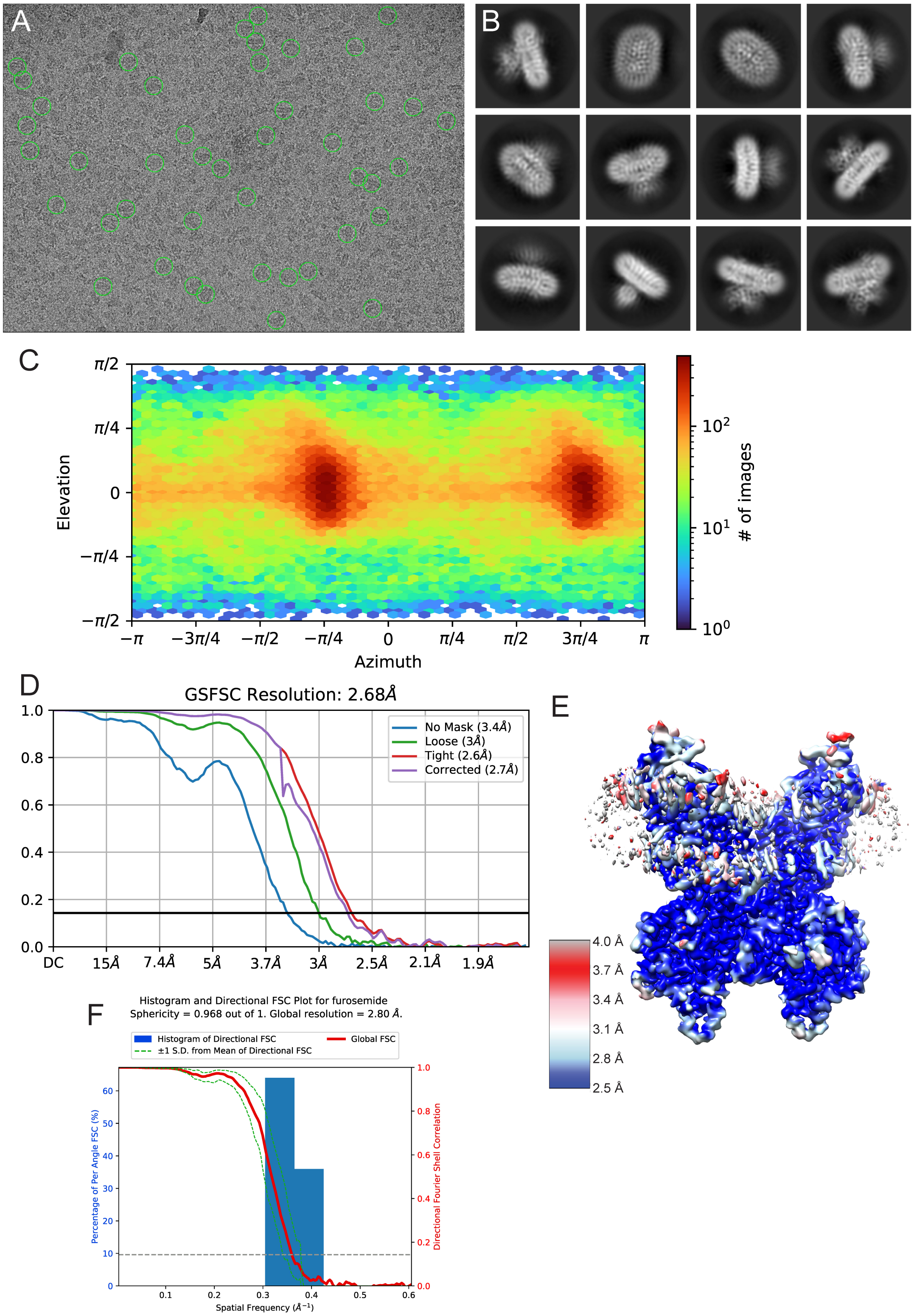
A
B
C
π/2
π/4
0
−π/4
−π/2
Elevation
−π
−3π/4
−π/2
−π/4
0
π/4
π/2
3π/4
π
Azimuth
10^2
10^1
10^0
# of images
D
GSFSC Resolution: 2.68Å
1.0
0.8
0.6
0.4
0.2
0.0
DC
15Å
7.4Å
5Å
3.7Å
3Å
2.5Å
2.1Å
1.9Å
No Mask (3.4Å)
Loose (3Å)
Tight (2.6Å)
Corrected (2.7Å)
E
4.0 Å
3.7 Å
3.4 Å
3.1 Å
2.8 Å
2.5 Å
F
Histogram and Directional FSC Plot for furosemide
Sphericity = 0.968 out of 1. Global resolution = 2.80 Å.
Histogram of Directional FSC
±1 S.D. from Mean of Directional FSC
Global FSC
Percentage of Per Angle FSC (%)
Directional Fourier Shell Correlation
Spatial Frequency (Å⁻¹)

Appendix Figure S3. Cryo-EM structure of human pNKCC1 bound with furosemide. (A) A representative micrograph of pNKCC1/furosemide recorded with a Titan Krios microscope. Representative particles are highlighted with 20 nm-diameter circles. (B) 2D class averages of pNKCC1/furosemide showed well-resolved structural features for the transmembrane and the cytosolic domains. (C) Angular distribution plot of all particle projections as output by cryoSPARC 3.0. (D) Gold-standard FSC curves calculated after cryoSPARC 3.0 non-uniform refinement. (E) Local resolutions calculated in cryoSPARC. (F) Directional resolutions were calculated using a remote 3DFSC processing server. Note, the final map shows reasonable sphericity (0.968) albeit the dataset exhibits uneven angular distribution.

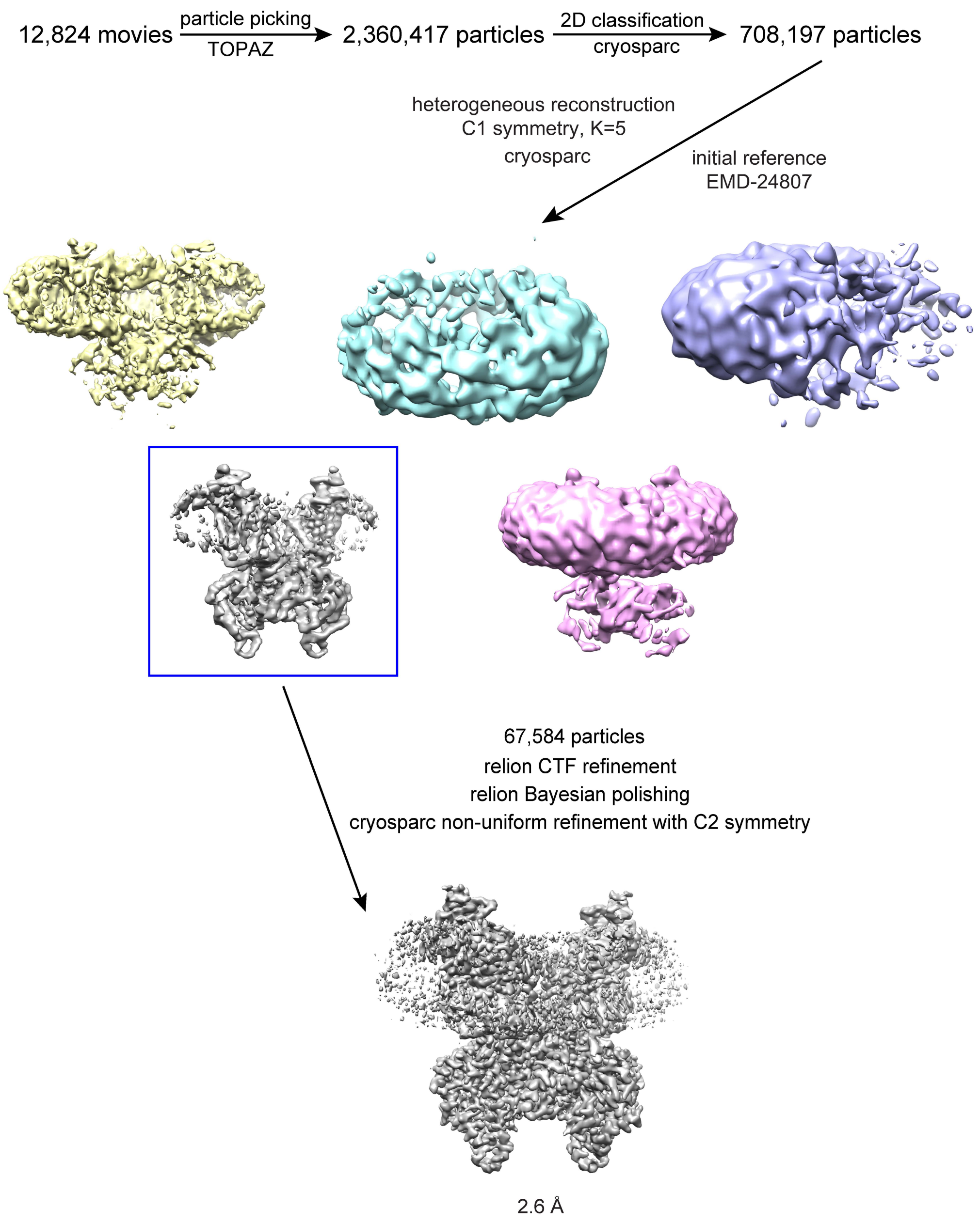

Appendix Figure S4. Reconstruction of human pNKCC1 bound with torsemide.
Flow chart of image processing for the pNKCC1/torsemide dataset.

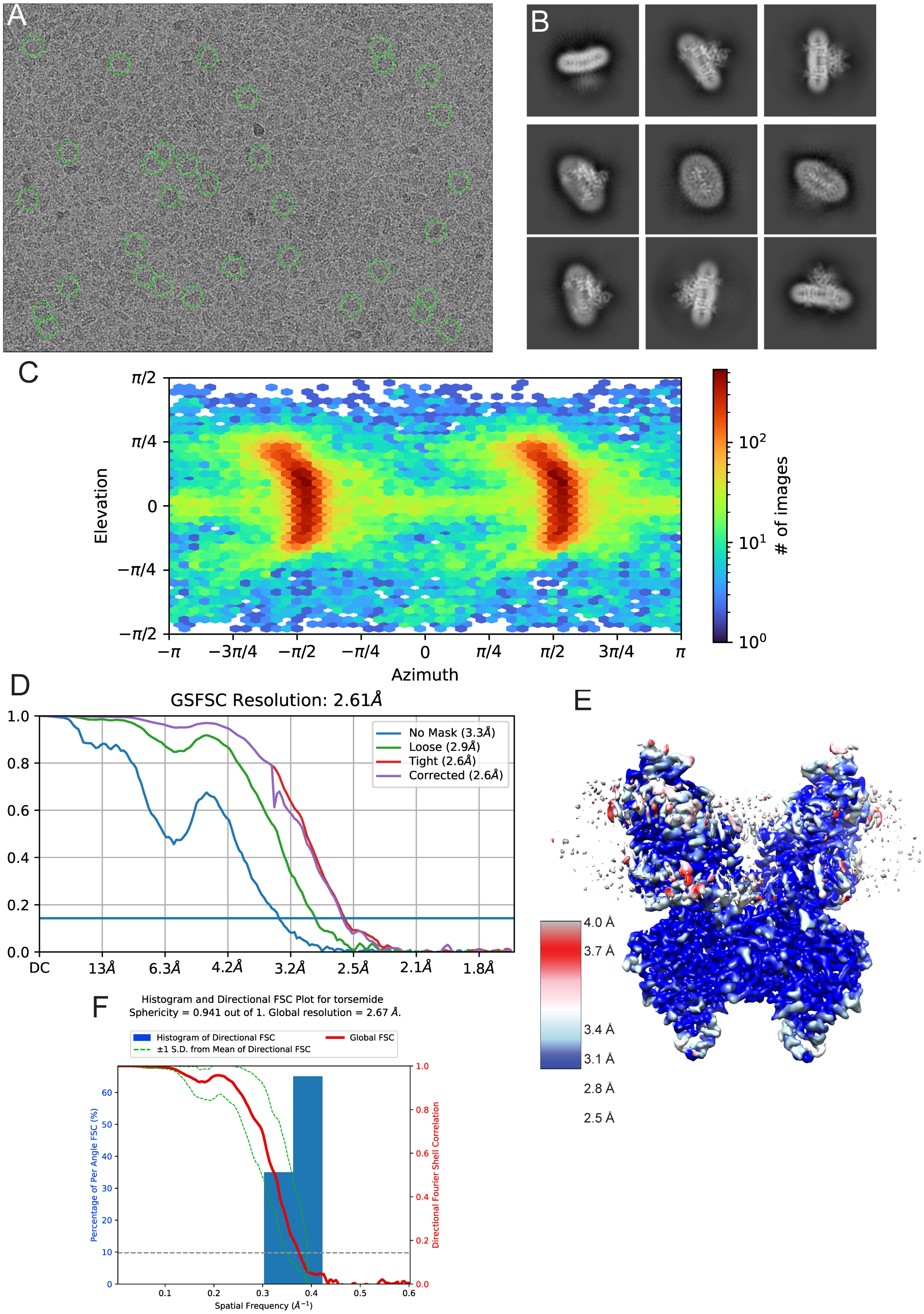
A
B
C
π/2
π/4
0
−π/4
−π/2
Elevation
−π
−3π/4
−π/2
−π/4
0
π/4
π/2
3π/4
π
Azimuth
10^2
10^1
10^0
# of images
D
GSFSC Resolution: 2.61Å
No Mask (3.3Å)
Loose (2.9Å)
Tight (2.6Å)
Corrected (2.6Å)
1.0
0.8
0.6
0.4
0.2
0.0
DC
13Å
6.3Å
4.2Å
3.2Å
2.5Å
2.1Å
1.8Å
E
4.0 Å
3.7 Å
3.4 Å
3.1 Å
2.8 Å
2.5 Å
F
Histogram and Directional FSC Plot for torsemide
Sphericity = 0.941 out of 1. Global resolution = 2.67 Å.
Histogram of Directional FSC
±1 S.D. from Mean of Directional FSC
Global FSC
Percentage of Per Angle FSC (%)
Directional Fourier Shell Correlation
Spatial Frequency (Å⁻¹)
0.1
0.2
0.3
0.4
0.5
0.6

Appendix Figure S5 Cryo-EM structure of human pNKCC1 bound with torsemide. (A) A representative micrograph of pNKCC1/torsemide recorded with a Titan Krios microscope. Representative particles are highlighted with 20 nm-diameter circles. (B) 2D class averages of pNKCC1/torsemide showed well-resolved structural features for the transmembrane and the cytosolic domains. (C) Angular distribution plot of all particle projections as output by cryoSPARC 3.0. (D) Gold-standard FSC curves calculated after cryoSPARC 3.0 non-uniform refinement. (E) Local resolutions calculated in cryoSPARC. (F) Directional resolutions were calculated using a remote 3DFSC processing server. Note, uneven angular distribution does not significantly cause anisotropy as indicated by a reasonable sphericity (0.941) of the final map.

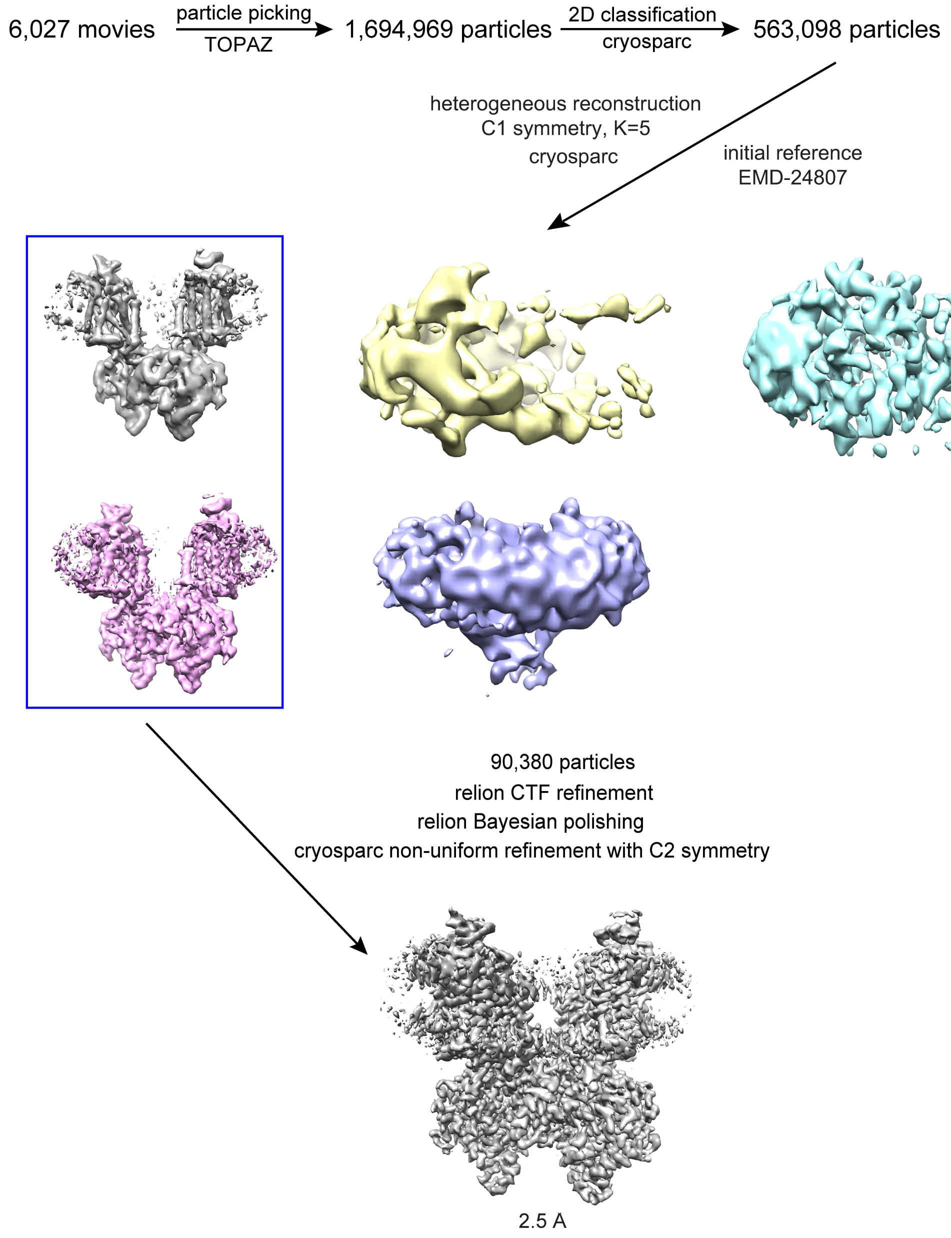

Appendix Figure S6. Reconstruction of human pNKCC1 bound with bumetanide.
Flow chart of image processing for the pNKCC1/bumetanide dataset.

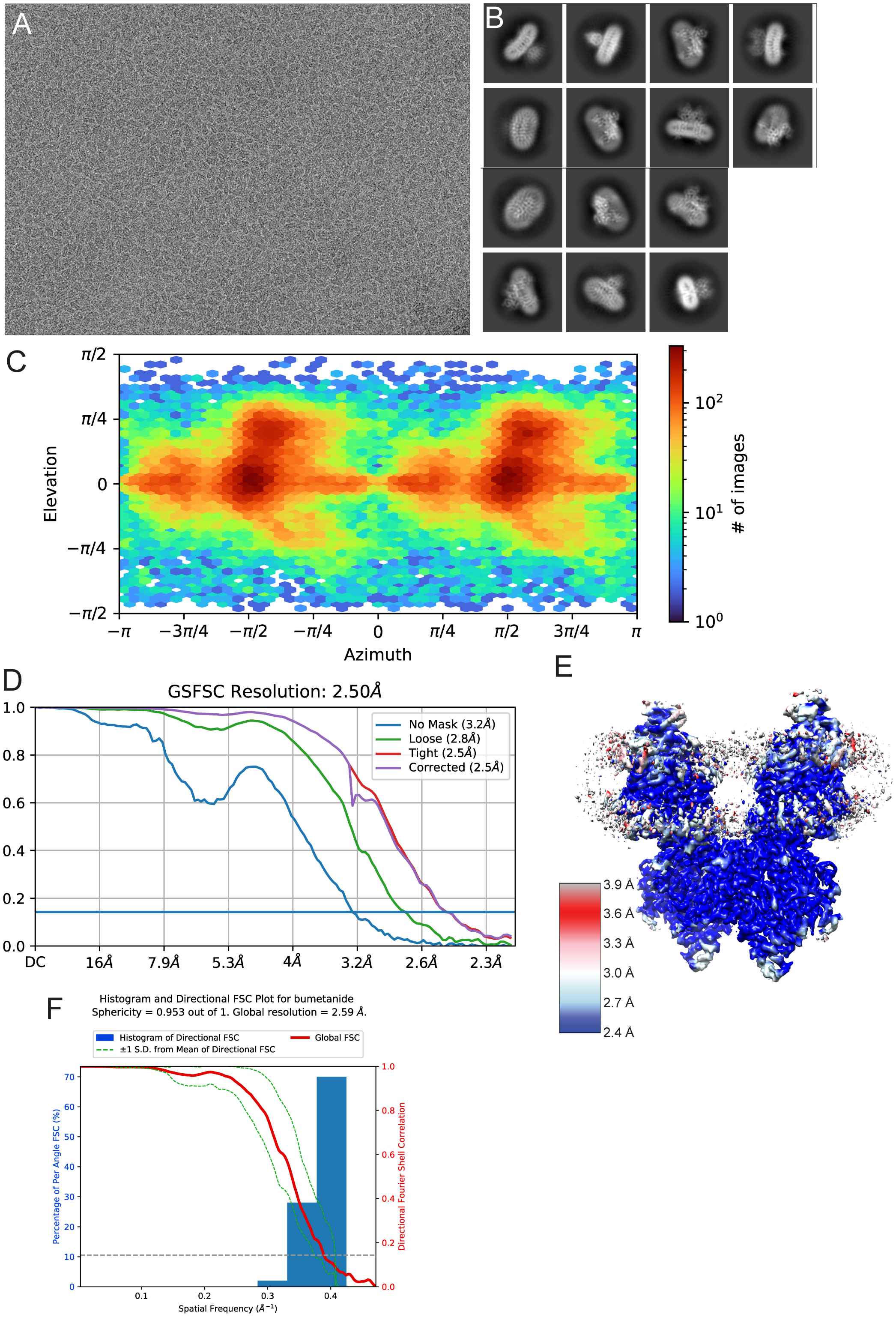
A
B
C
Elevation
Azimuth
# of images
D
GSFSC Resolution: 2.50Å
No Mask (3.2Å)
Loose (2.8Å)
Tight (2.5Å)
Corrected (2.5Å)
DC
16Å
7.9Å
5.3Å
4Å
3.2Å
2.6Å
2.3Å
E
3.9 Å
3.6 Å
3.3 Å
3.0 Å
2.7 Å
2.4 Å
F
Histogram and Directional FSC Plot for bumetanide
Sphericity = 0.953 out of 1. Global resolution = 2.59 Å.
Histogram of Directional FSC
±1 S.D. from Mean of Directional FSC
Global FSC
Percentage of Per Angle FSC (%)
Directional Fourier Shell Correlation
Spatial Frequency (Å⁻¹)

Appendix Figure S7. Cryo-EM structure of human pNKCC1 bound with bumetanide. (A) A representative micrograph of pNKCC1/bumetanide recorded with a Titan Krios microscope. (B) 2D class averages of pNKCC1/bumetanide showed well-resolved structural features for the transmembrane and the cytosolic domains. (C) Angular distribution plot of all particle projections as output by cryoSPARC 3.0. (D) Gold-standard FSC curves calculated after cryoSPARC 3.0 non-uniform refinement. (E) Local resolutions calculated in cryoSPARC. (F) Directional resolutions were calculated using a remote 3DFSC processing server.

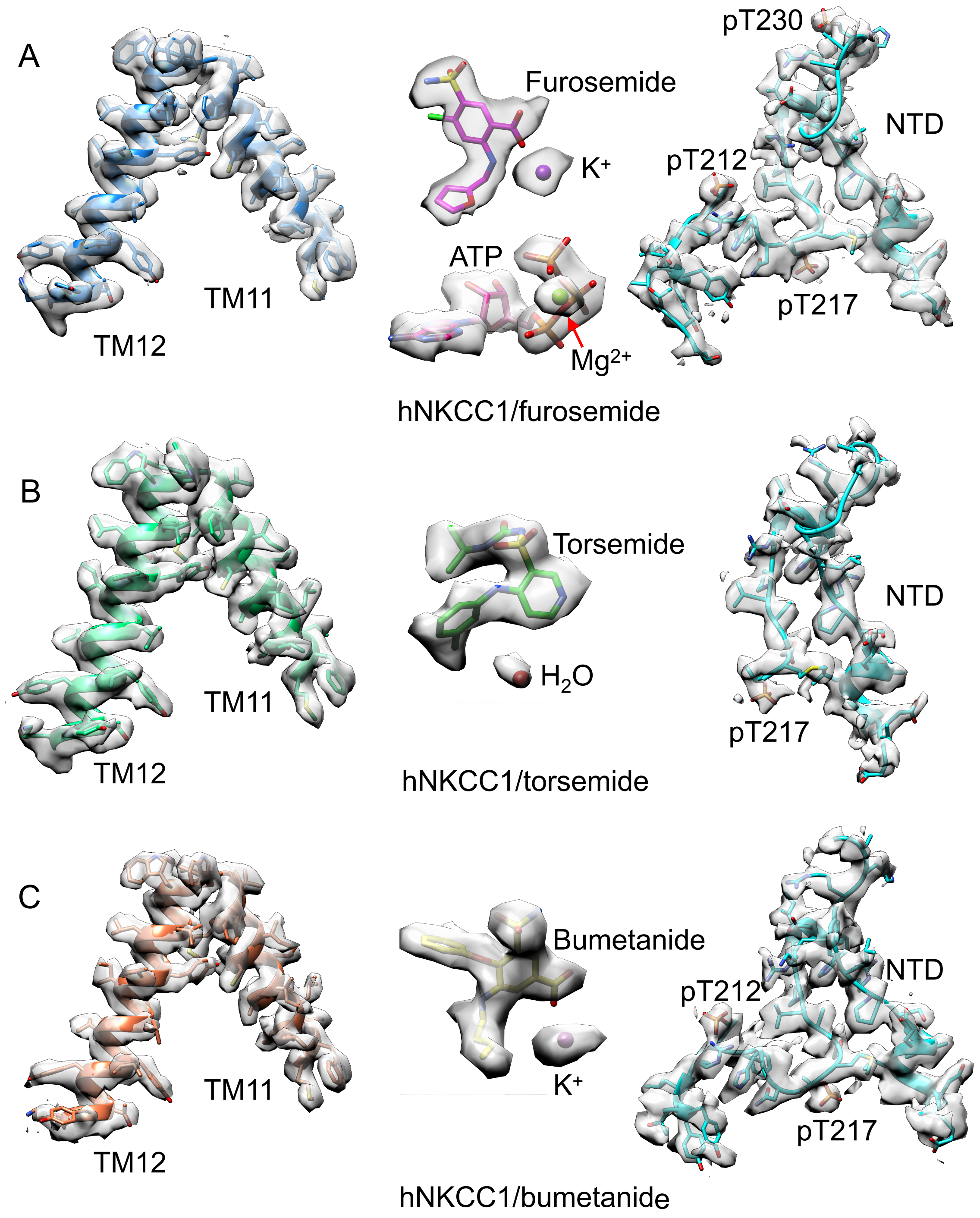

A
Furosemide
K+
ATP
Mg2+
TM11
TM12
pT230
NTD
pT212
pT217
hNKCC1/furosemide
B
Torsemide
H2O
TM11
TM12
NTD
pT217
hNKCC1/torsemide
C
Bumetanide
K+
TM11
TM12
NTD
pT212
pT217
hNKCC1/bumetanide

Appendix Figure S8. EM density maps of representative regions from the pNKCC1/furosemide, pNKCC1/torsemide, and pNKCC1/bumetanide structures. The final models shown in stick are docked into experimental densities. (A) The EM density maps of representative regions from pNKCC1/furosemide. (B) The EM density maps of representative regions from pNKCC1/torsemide. (C) The EM density maps of representative regions from pNKCC1/bumetanide. Data information: The representative EM densities of pNKCC1/furosemide, pNKCC1/torsemide, and pNKCC1/bumetanide were extracted from original sharpen maps and displayed at a contour level of 0.218, 0.203, 0.224 in UCSF Chimera, respectively.

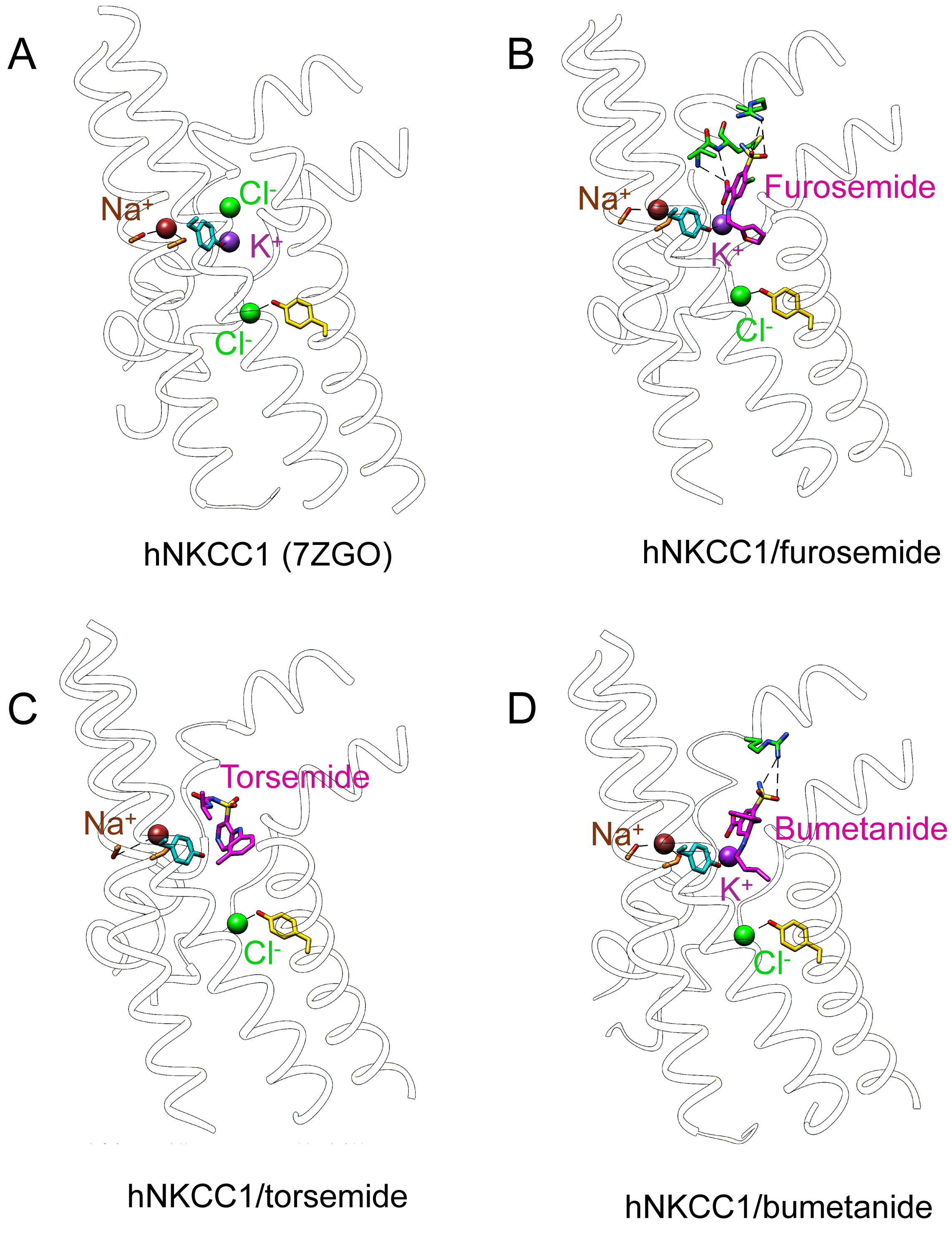
A
Na+
Cl-
K+
Cl-
hNKCC1 (7ZGO)
B
Na+
Furosemide
K+
Cl-
hNKCC1/furosemide
C
Torsemide
Na+
Cl-
hNKCC1/torsemide
D
Na+
Bumetanide
K+
Cl-
hNKCC1/bumetanide

Appendix Figure S9. Furosemide, torsemide, and bumetanide bind to an orthosteric site shared with the substrate ions. (A) The substrate ions in NKCC1 structure (PDB code: 7ZGO). (B) The substrate ions and furosemide in pNKCC1/furosemide structure. (C) The substrate ions and furosemide in pNKCC1/torsemide structure. (D) The substrate ions and furosemide in pNKCC1/bumetanide structure. The K^+, Na^+, Cl^-, and bumetanide are colored in purple, brown, green, and magenta, respectively. The key ion coordinating residues S613, S614, Y383, and Y686 are highlighted in stick.

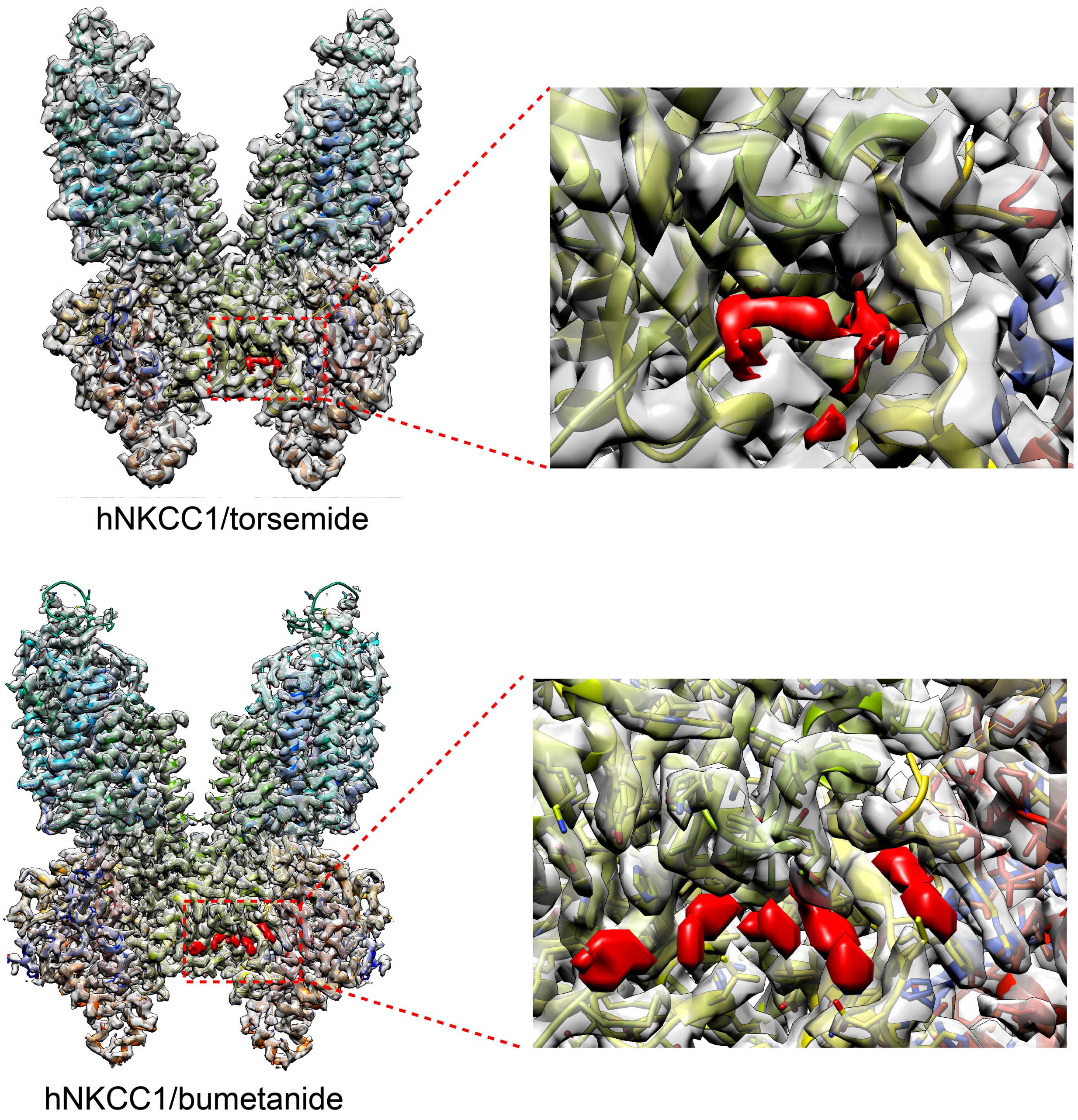

Appendix Figure S10. Non-protein densities in the amphipathic ATP-binding pocket in the CTD of pNKCC1/torsemide and pNKCC1/bumetanide maps. The potential densities for ATP are shown in red.

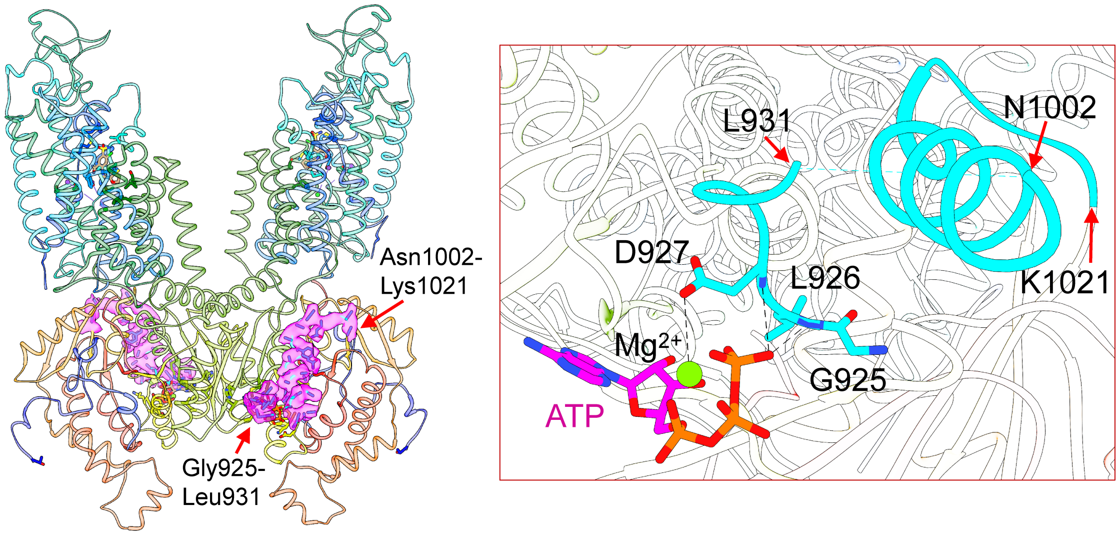

Appendix Figure S11. ATP-binding stabilizes the Gly925-Leu931 and Asn1002-Lys1021 segments of NKCC1. Mg^{2+} is shown as a green sphere.

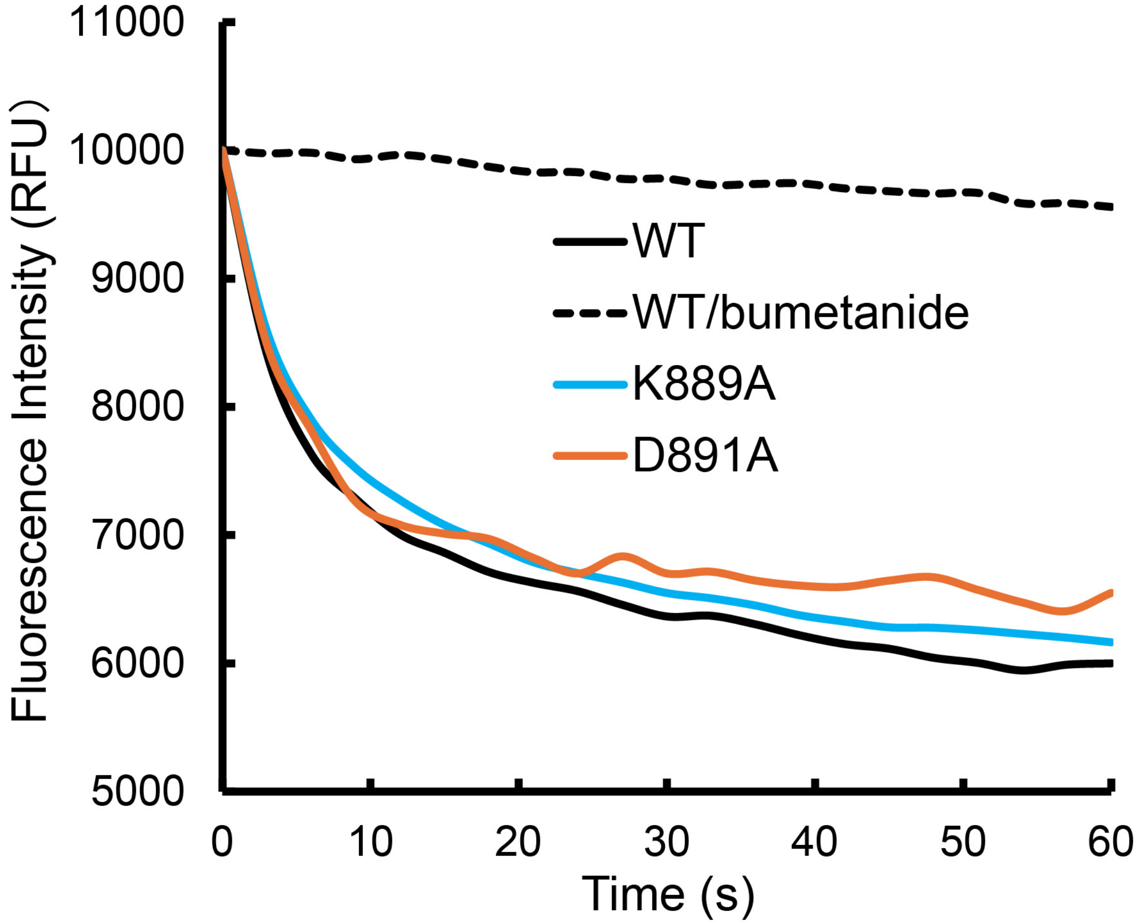

Appendix Figure S12. Weakening the phosphate-mediated ATP binding by substitution of Lys889 or Asp891 to alanine doesn't affect ion transport rate of NKCC1 (n=6).

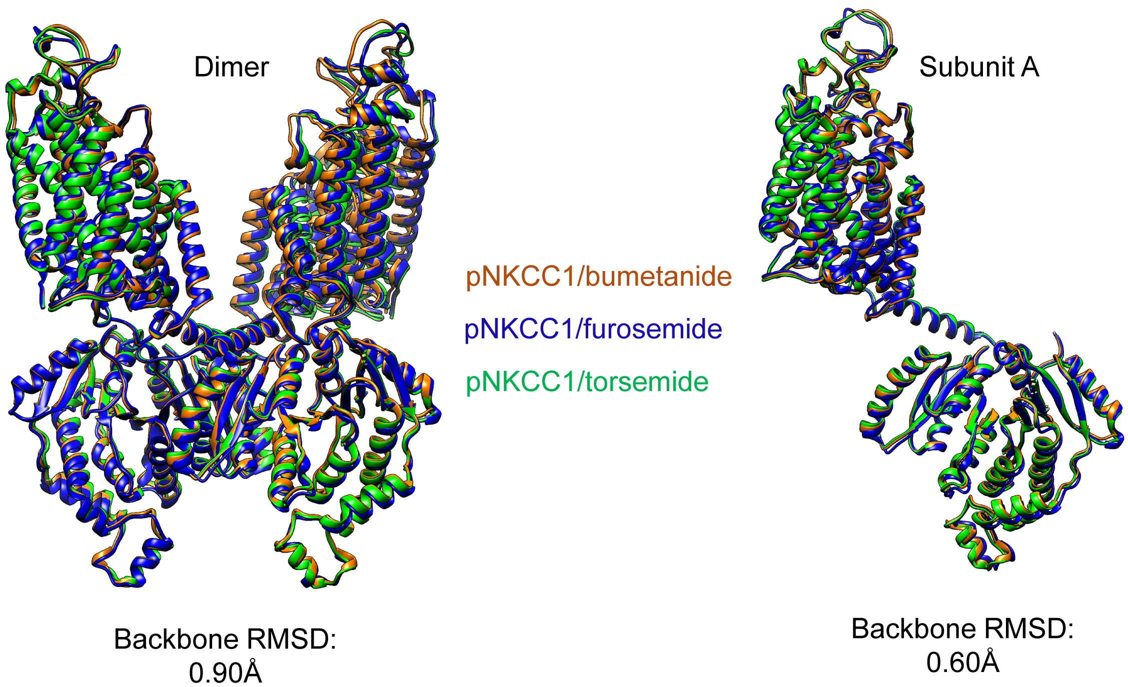

Appendix Figure S13. Comparison of structures of pNKCC1/bumetanide, pNKCC1/furosemide, and pNKCC1/torsemide. RMSD among pNKCC1/bumetanide, pNKCC1/furosemide, and pNKCC1/torsemide was calculated using VMD program (Humphrey W, Dalke A, Schulten K. VMD: visual molecular dynamics. *J Mol Graph* **14:** 33-38, 27-38)

Appendix Table S1 | Statistics of cryo-EM data acquisition and processing and model building

**Data collection/Processing**			
	NKCC1/ furosemide	NKCC1/ torsemide	NKCC1/ bumetanide
Microscope	Titan Krios K3 camera	Titan Krios K3 camera	Titan Krios K3 camera
Voltage (kV)	300	300	300
Defocus range (μm)	-0.7 – -3.5	-0.7 – -3.5	-0.7 – -3.5
Pixel size (Å)	0.8256	0.83	1.06
Total electron dose ($e^-/Å^2$)	50	42	60
Exposure time (s)	2.5	2.5	2.5
Number of movies	14,999	12,824	6,027
Number of frames per movie	38	50	40
Initial particle number	8,051,818	2,360,417	1,694,969
Final particle number	118,697	67,584	90,380
Resolution (unmasked, Å)	3.4	3.3	3.2
Resolution (masked, Å)	2.68	2.6	2.5
**Refinement and Validation**			
Number of atoms	13,584	12,990	13,126
R.M.S deviation			
Bond length (Å)	0.002	0.003	0.004
Bond angles (°)	0.439	0.514	0.639
**Ramachandran**			
Favored (%)	95.69%	94.61%	93.68%
Allowed (%)	4.31%	5.39%	6.32%
Outlier (%)	0.00%	0.00%	0.00%
MolProbity score	2.63	2.72	2.35
