# Supplementary material for: Structural basis for human NKCC1 inhibition by loop diuretic drugs
Source: EMBO J. 2025 Jan 28;44(5):1540–62. doi: 10.1038/s44318-025-00368-6 (PMC11876703; doi:10.1038/s44318-025-00368-6)
Supplement: Supplementary file 7 — Expanded View Figures [file 44318_2025_368_MOESM7_ESM.pdf]

Expanded View Figures

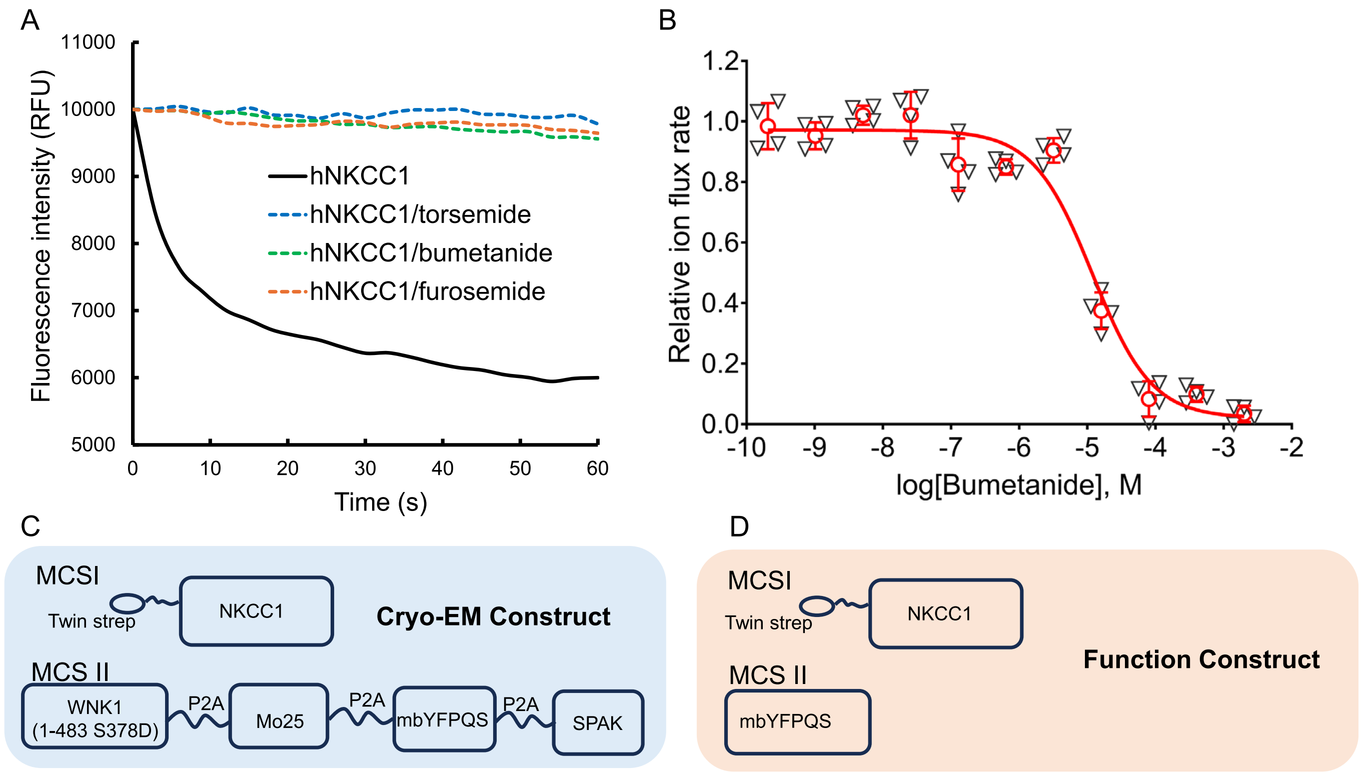

Figure EV1. Functional characterization of human NKCC1 in HEK293 cells.

(**A**) NKCC1-mediated Cl^- influx was inhibited by furosemide, bumetanide, and torsemide applied externally to the cells at 100 μM concentration. (**B**) Dose-response curve was determined for NKCC1 inhibition by bumetanide. Data information: Each triangle represents one kinetic measurement of a single sample incubated with bumetanide of indicated concentration in a 96-well plate ($n = 4$ biological repeats; data are presented as mean values $+/-$ SD). The dose-response curve is fitted to the standard equation of log[bumetanide] versus response (three parameters) using GraphPad Prism 8.0. (**C**) Design of NKCC1 constructs for cryo-EM studies. (**D**) Design of NKCC1 constructs for functional studies.

 © The Author(s)

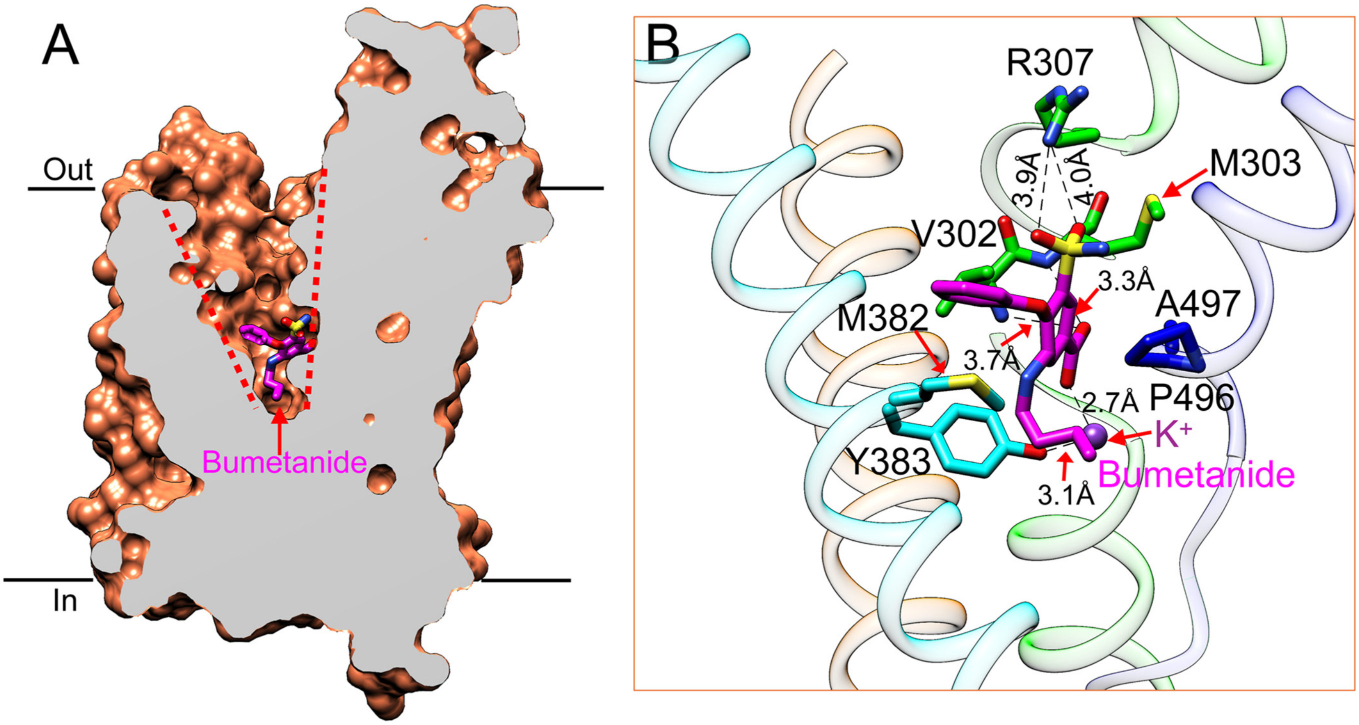

Figure EV2. Bumetanide binds to the NKCC1 extracellular vestibule and traps it in an outward-open state.

(**A**) A "cut-off" view of pNKCC1/bumetanide co-structure highlights an extracellular vestibule in which bumetanide resides. (**B**) A view of bumetanide binding pocket highlights the key coordinating residues.

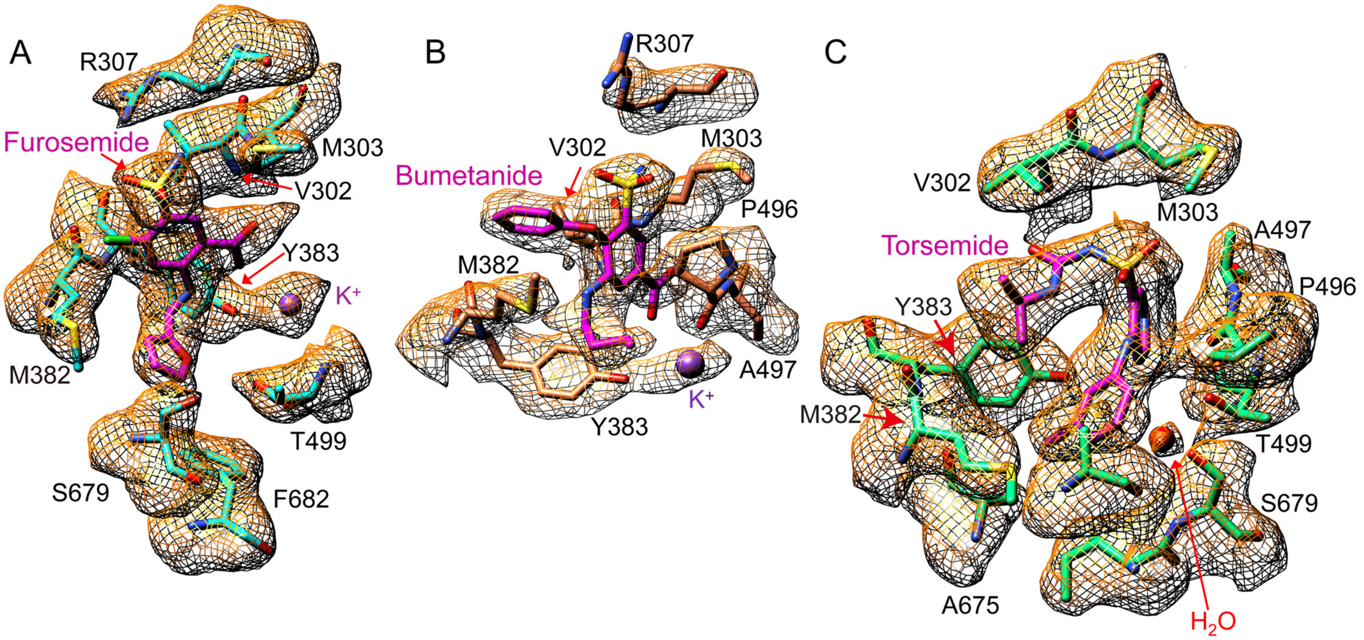

Figure EV3. Chemical environment of loop diuretics binding sites.

(**A**) Well-resolved furosemide, K^+, and their coordinating residues were docked into cryo-EM densities. Data information: The densities for furosemide, K^+, and its coordinating residues were extracted from original sharpened map and displayed at a contour level of 0.202 in UCSF Chimera. (**B**) Well-resolved bumetanide, K^+, and their coordinating residues were docked into cryo-EM densities. Data information: The densities for bumetanide, K^+, and its coordinating residues were extracted from original sharpened map and displayed at a contour level of 0.241 in UCSF Chimera. (**C**) Well-resolved torsemide, water, and their coordinating residues were docked into cryo-EM densities. Data information: The densities for torsemide, water, and their coordinating residues were extracted from original sharpened map and displayed at a contour level of 0.141in UCSF Chimera.

 © The Author(s)

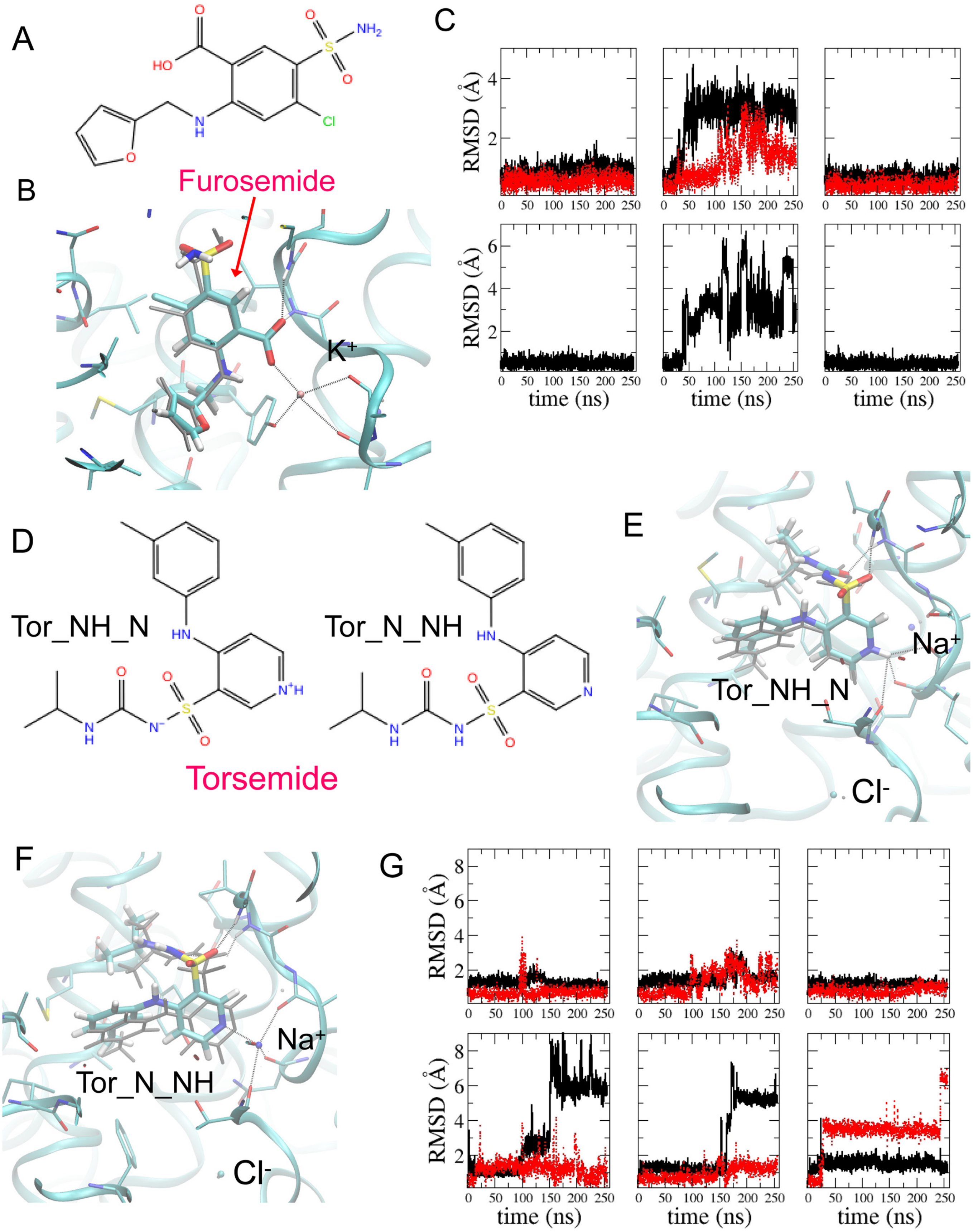
A
HO
O
NH2
Cl
N
H
B
Furosemide
K+
C
RMSD (Å)
time (ns)
D
Tor_NH_N
Tor_N_NH
Torsemide
E
Na+
Tor_NH_N
Cl-
F
Tor_N_NH
Na+
Cl-
G
RMSD (Å)
time (ns)

Figure EV4. Molecular dynamics simulations of furosemide and torsemide when bound to NKCC1.

(**A**) Chemical structure of furosemide. (**B**) Structure after 250 ns simulation of furosemide; the initial experimental pose of furosemide and K^+ are shown in gray. (**C**) Root mean square displacement (RMSD) with respect to the initial pose of furosemide (black line) and K^+ ion (red dotted line) showed in the top three panels and of Na^+ showed in the bottom three panels. Three replicas of the NKCC1/furosemide complex were simulated. Note, in the second simulation (the two middle panels), the high mobility of Na^+ likely also causes instability of furosemide in the pocket. (**D**) Chemical structures of two possible forms of torsemide in neutral pH are shown. (**E**) Structure after 250 ns simulation of Tor_NH_N; the initial pose of torsemide, Na^+ and Cl^- are shown in gray. (**F**) Structure after 250 ns simulation of Tor_N_NH; the initial pose of torsemide, Na^+ and Cl^- are shown in gray. (**G**) RMSD with respect to the initial pose of torsemide (black line) and Na^+ ion (red line) for Tor_NH_N (top panels) and Tor_N_NH (bottom panels). For each protonation state, three replicas were simulated.

© The Author(s)

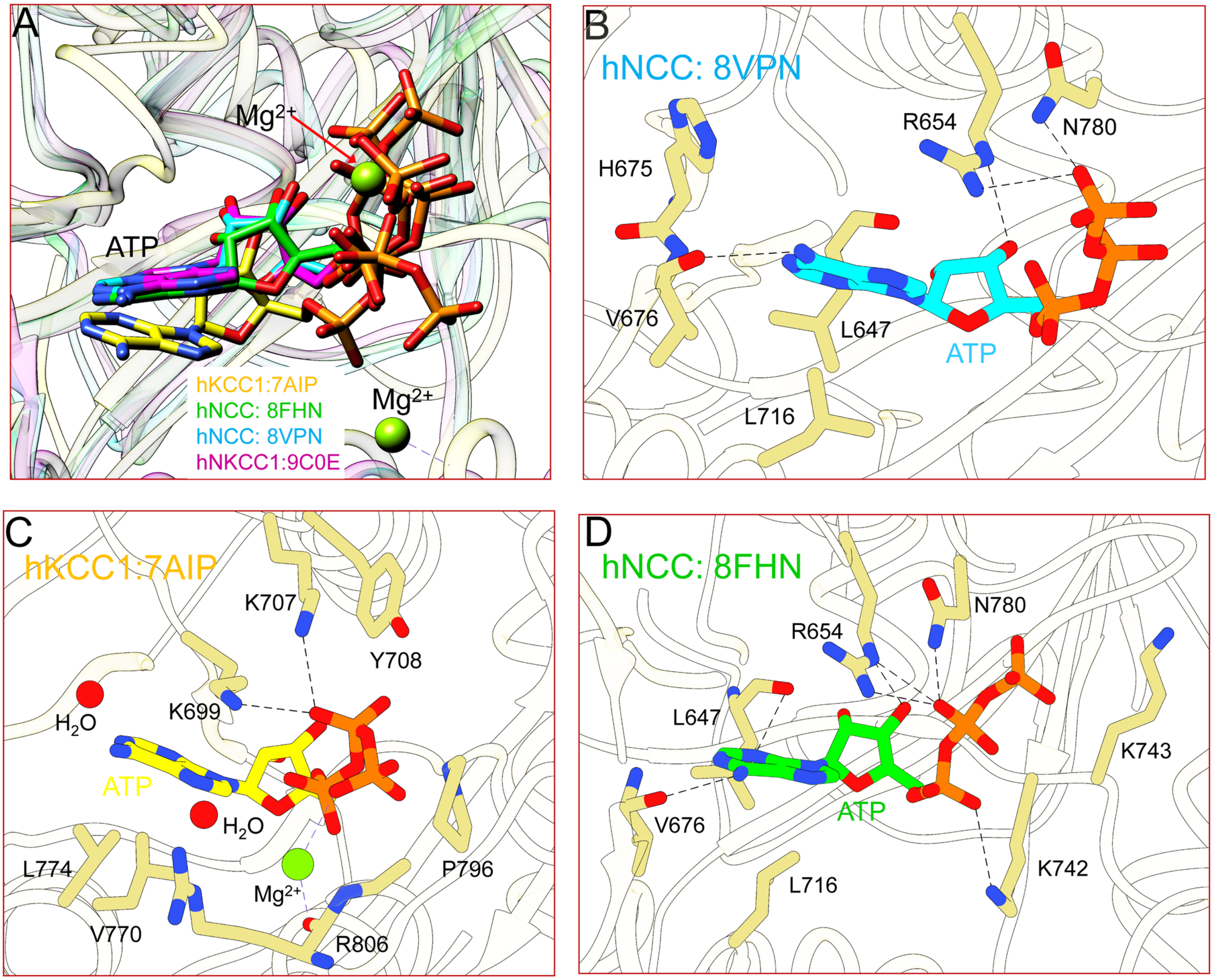

Figure EV5. Comparison of ATP poses and its coordinating residues in NKCC1, NCC, and KCC1.

(**A**) A superimposition of ATP in human KCC1 (PDB code: 7AIP), human NCC (PDB code: 8VPN and 8FHN) and human NKCC1 (PDB code: 9C0E). (**B**) The ATP and coordinating residues in human NCC (PDB code: 8VPN) were highlighted in sticks. (**C**) The ATP and coordinating residues in human KCC1 (PDB code: 7AIP) were highlighted in sticks. (**D**) The ATP and coordinating residues in human NCC (PDB code: 8FHN) were highlighted in sticks.

© The Author(s)
